# Unexpected little helpers: microbiota stimulate systemic antiviral defenses

**DOI:** 10.1038/s41392-022-01217-2

**Published:** 2022-10-15

**Authors:** Susanne Klute, Helene Hoenigsperger, Konstantin M. J. Sparrer

**Affiliations:** Institute of Molecular Virology, Ulm University Medical Center, Ulm, 89081 Germany

**Keywords:** Microbiology, Innate immunity

In a recent study published in *Immunity* by Erttmann et al., an unexpected beneficial role of gut microbiota in protecting mice against invading viruses by promoting low levels of systemic innate immune activation via type I interferon (IFN) priming was revealed.^1^

This adds to accumulating evidence of a positive impact of balanced gut microbiota on shaping and training both the adaptive and innate immune responses.^2^ In turn, the immune system maintains and regulates this delicate, symbiotic balance. However, the underlying molecular mechanisms are poorly understood. Innate immune activation by microbiota is mediated by a dedicated, germ-line encoded set of innate immune sensors, so-called pattern recognition receptors. Microbials breaching the barriers of the gut epithelium are classically sensed by Toll-like receptors (TLRs). Subsequent inflammatory responses provoke local antibacterial immune responses but also cause and/or contribute to the pathogenesis of many diseases.^2^ However, the recent study by Erttmann et al. showed that non-invading commensal bacteria activated beneficial systemic type I IFN innate immune responses, independent of TLRs and direct bacteria-cell contacts. Removal or disturbance of the gut microbiota by antibiotic treatment reduced overall type I IFN induction in vivo. Conversely, fecal transplants after antibiotic treatment restored levels of immune activation. The authors show that membrane vesicles (MVs) derived from Gram-negative and Gram-positive gut bacteria (Fig. 1) were sufficient to sustain a low-level type I IFN response. Production of MVs is an integral part of many bacteria (including common commensals) regulating bacterial functions such as quorum sensing, biofilm formation, antibiotic resistance, and nutrient acquisition. Agarose gel analysis and 16 S sequencing revealed the presence of DNA derived from various bacterial phyla within the MVs, suggesting that these vesicles deliver bacterial DNA upon fusion with host cells. The main sensor for cytoplasmic DNA is the cyclic GMP–AMP synthase (cGAS). Upon activation, cGAS produces 2’–3’ linked cyclic GTP-ATP (cGAMP). cGAMP in turn binds to the stimulator of interferon genes (STING), which oligomerizes, and is transported from the endoplasmic reticulum (ER) to the Golgi. Activation of STING eventually leads to transcriptional upregulation and secretion of type I IFNs and other pro-inflammatory cytokines (Fig. 1). The secreted IFNs bind in an auto- or paracrine fashion to cells, inducing transcription of hundreds of IFN stimulated genes (ISGs), setting the cells into an antimicrobial state.^3^ Consistent with the role of the cGAS-STING axis, bacteria-derived MVs failed to stimulate STAT transcription factor activation in STING KO cells, but not upon KO of signaling adaptors of other innate immune sensors. In line with this, reduced basal priming of the type I IFN response was observed in STING knockout mice and bone marrow-derived mouse macrophages (BMDMs) deficient in cGAS. To exclude that TLR-dependent detection of microbiota contributes to elevated type I IFN levels, the authors used a set of knockout mice, deficient in TLR and/or STING. Their data shows a significant reduction of basal type I IFN levels only for the STING knockout mice indicating only a minor role of TLRs in systemic priming. Notably, the authors show that MVs containing bacterial DNA can pass the gut epithelial barrier. Consequently, these MVs were also detected in feces as well as in the blood serum of mice, indicating induction of a cGAS-dependent type I IFN beyond a local response. Analyses of the spatial distribution of *ifnb1* levels in mice after *E. coli* or *E. coli*-derived MV priming also support a systemic elevation of type I IFNs. Again, this systemic response is abrogated in cGAS KO mice. As a consequence of low level systemic type I IFN induction, antiviral defenses are primed, and replication of viral pathogens like the DNA virus Herpes simplex virus 1 (HSV-1) or the RNA virus Vesicular stomatitis virus (VSV) is reduced in mice with a balanced microbiota (Fig. 1). Disturbance of the microbiota by antibiotic treatment lead to enhanced replication of both viruses. Of note, VSV is not sensed by cGAS, but cGAS- or STING-deficient mice show increased replication of VSV, additionally confirming the important role of microbiota-driven cGAS-STING activation in enhancing antiviral defenses.

**Fig. 1:**
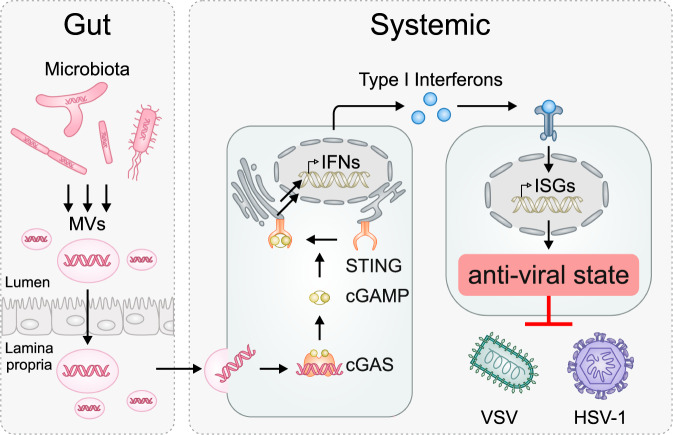
Activation of cGAS-STING-dependent type I IFN signaling via DNA-containing membrane vesicles released by gut microbiota. Bacterial DNA-containing MVs are released by the microbiota in the gut and cross the epithelial barrier. In MV-targeted cells, the DNA is recognized by cGAS, which synthesizes the second messenger cGAMP. cGAMP binds and induces oligomerization and translocation of STING from the ER to the Golgi, eventually causing induction of type I IFNs. Secreted IFNs are recognized by IFN-receptors resulting in the activation of a signaling cascade leading to the induction of hundreds of ISGs, which set cells in an antiviral state. MVs membrane vesicles, IFN interferon, cGAS cyclic GMP–AMP synthetase, cGAMP cyclic guanosine monophosphate–adenosine monophosphate, STING stimulator of interferon genes, ISG interferon-stimulated gene, VSV Vesicular stomatitis virus, HSV-1 Herpes simplex virus 1

In summary, the study by Erttmann et al. demonstrates a role of microbiota in priming systemic type I IFN responses via the cGAS-STING axis, thereby protecting local and distal host cells from external threats: viruses. This conceptual framework that microbiota promote antiviral immunity is supported by mechanistic evidence highlighting commensal-derived MVs as a systemic communication platform between the microbiota and the innate immune system. Although microbiota-derived MVs were previously suggested to mediate host–microbe interactions, their mode of action had been unclear so far. As this study highlights new mechanistic aspects of the mutually beneficial relationship between commensal bacteria and their host, interesting questions are prompted. Is the situation in humans similar to mice? How systemic is the MV-induced type I IFN response, and which tissues/cell types are affected? Would elevated type I IFN levels also affect pathogenic bacteria and to some extent a local response also target the gut microbiota themselves? Is there an evolutionary advantage for bacteria to promote the antiviral capacities of their host? Of note, the other way around—viruses being beneficial in host immune reactions—has already been shown. Herpesviruses were reported to protect mice against pathogenic *Listeria monocytogenes* infection.^4^ Pathology of many diseases is driven by elevated levels of inflammatory IFNs, most prominently including a set of monogenic inflammatory diseases called type I interferonopathies.^5^ Whether and how the low level “chronic”, but beneficial inflammation induced by bacterial MVs are tolerated in the long run remains unknown. It is tempting to speculate that these elevated levels of systemic type I IFN are typically below a pathogenic threshold, thus avoiding detrimental effects. This begs the question how the microbiota or the host regulates and limits this immune activation. Most importantly, the conclusions of this study may need to be considered for current antibacterial therapies using broad-spectrum antibiotics. Erttmann and colleagues suggest that disturbance or depletion of balanced gut microbiota by antibiotics may—besides other health issues—also convey an elevated risk of viral infections.

## References

[1] Erttmann SF (2022). The gut microbiota prime systemic antiviral immunity via the cGAS-STING-IFN-I axis. Immunity.

[2] Zheng D, Liwinski T, Elinav E (2020). Interaction between microbiota and immunity in health and disease. Cell Res..

[3] Sparrer KMJ, Gack MU (2015). Intracellular detection of viral nucleic acids. Curr. Opin. Microbiol..

[4] Barton ES (2007). Herpesvirus latency confers symbiotic protection from bacterial infection. Nature.

[5] Crow YJ, Stetson DB (2022). The type I interferonopathies: 10 years on. Nat. Rev. Immunol..

